# Thermoreversible Biogels for Intranasal Delivery of Rizatriptan Benzoate

**Published:** 2009

**Authors:** Renuka Chand, Anuja A. Naik, Hema A. Nair

**Affiliations:** Department of Pharmaceutics, Bombay College of Pharmacy, Kalina, Santacruz (E), Mumbai-400 098, India

**Keywords:** Thermoreversible gels, rizatriptan benzoate, chitosan

## Abstract

The objective of the present study was to formulate and evaluate a thermoreversible formulation containing rizatriptan benzoate for intranasal administration. Chitosan and aqueous *β*-glycerolphosphate were mixed in cold condition to obtain chitosan-*β*-glycerolphosphate mixtures, which served as the thermoreversible systems. Rizatriptan benzoate was incorporated at a final strength of 25 mg/ml. Both *in vitro* release and *ex vivo* permeation of rizatriptan from gels were measured at 37° using Franz diffusion cells Formulations were tested *in vivo* in mice for reduction in locomotor activity using digital actophotometer and nasal mucosal tissues were examined histopathologically.

The nasal route has been successfully exploited for systemic delivery of drugs and vaccines. This route also offers the possibility of preferential targeting of drugs to CNS via olfactory pathway, bypassing the blood brain barrier[1]. The objective of the present study was to formulate and evaluate a thermoreversible formulation containing the antimigraine drug rizatriptan benzoate (RB) for intranasal (IN) administration. The gels are based on the mucoadhesive biopolymer chitosan and utilize β–glycerolphosphate (GP) (C-GP-PEG) and without PEG (polyethylene glycol) (C-GP).

## MATERIALS AND METHODS

Chitosan (degree of deactylation∼89%) and RB were gifted by CIFT and Cipla Pvt. Ltd. respectively. GP was purchased from CDH and PEG 4000 from S. D. Fine Chem, Mumbai, India. All other reagents used in the study were of analytical grade.

### Preparation of gels:

Chitosan dissolved in 0.1N HCl and aqueous GP were mixed in cold condition to obtain chitosan-GP (C-GP) mixtures, which served as the thermoreversible systems. Formulations containing PEG 1% w/v (C-GP-PEG) with lower GP content were also prepared. RB was incorporated at a final strength of 25 mg/ml.

### Evaluation of Gels:

The gelling temperature and time were measured by gradually warming the sols until movement of the meniscus was arrested. Gel strength was measured at 37° in terms of time taken for a 7 g stainless steel ball to fall through 4 cm height of gel. Both *in vitro* release and *ex vivo* permeation of RB from gels into PBS (pH 6.4) were measured at 37° using Franz diffusion cells across parchment paper and sheep nasal mucosa, respectively followed by UV spectrophotometric analysis[2]. Mucoadhesive strength of the formulations in both sol and gel states were determined using a modified two-pan balance as the force required to separate two porcine mucin coated surfaces with gel between them. Formulations were tested *in vivo* in mice for reduction in locomotor activity using digital actophotometer and nasal mucosal tissues were examined histopathologically. Statistical analysis was performed using ANOVA followed by Bonferroni's multiple comparison test whenever applicable (P value<0.001).

## RESULTS AND DISCUSSION

**Fig. 1:**
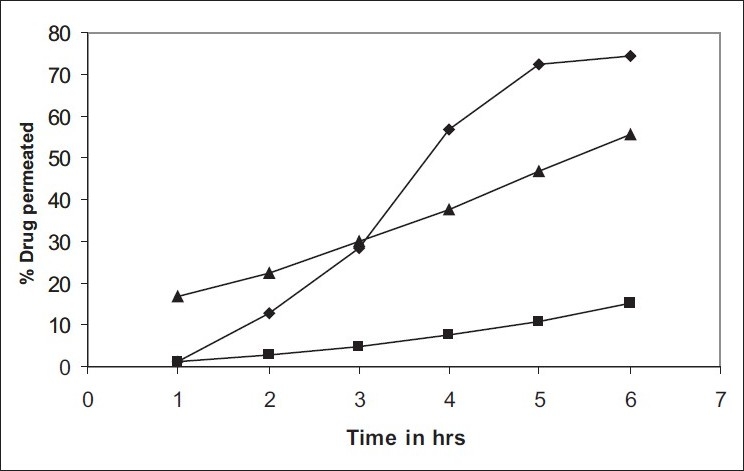
*Ex vivo* permeation studies *Ex vivo* permeation studies of RB from solution and from thermoreversible formulations, (–◆–) C-GP, (–▪–) C-GP-PEG and (–▲–) drug solution

**Fig. 2:**
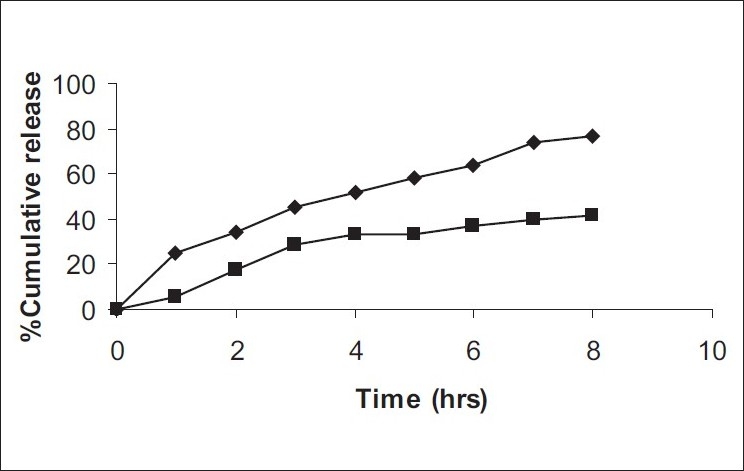
*In vitro* drug release studies *In vitro* drug release of RB from thermoreversible formulations. (–◆–) C-GP and (–▪–) C-GP-PEG

**Fig. 3:**
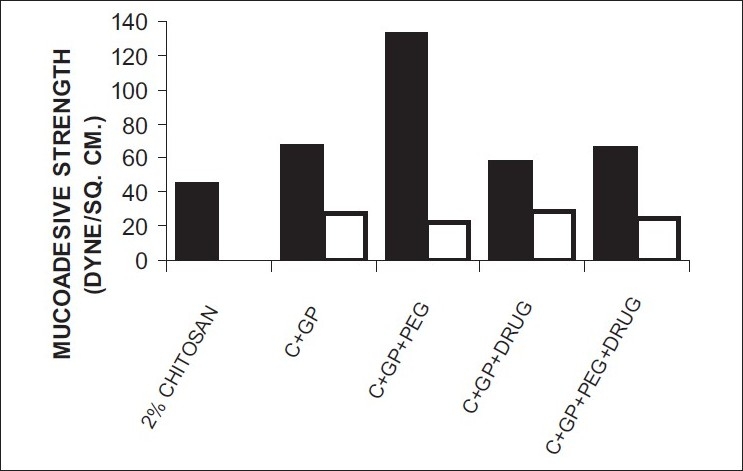
Mucoadhesive strength Mucoadhesive strength of formulations at (▪) 25° and (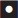) 37°

**Fig. 4:**
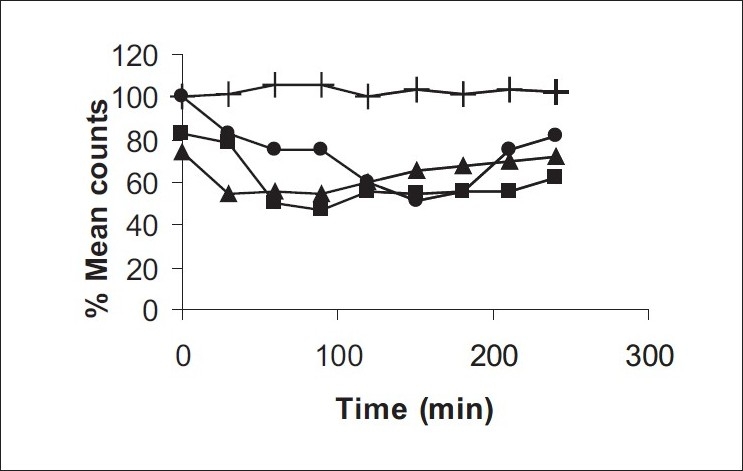
Locomotor activity of mice after intranasal administration Locomotor activity of mice after intranasal administration of rizatriptan benzoate. (–+–) control, (–▪–) C-GP, (–•–) C-GP-PEG and (–▲–) drug solution

**Fig. 5:**
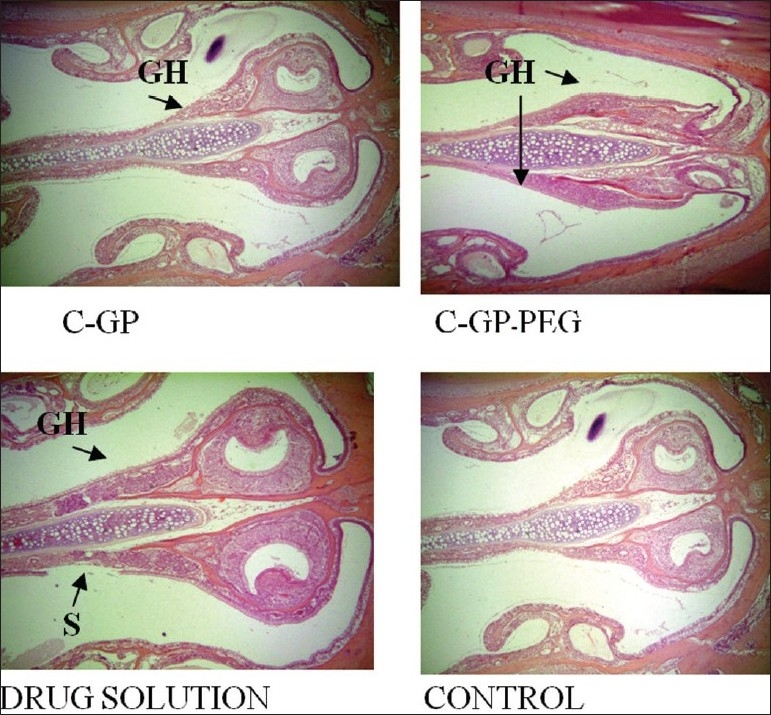
Histopathology of nasal mucosa after 5 days of exposure S: Sluffing of nasal mucosa, GH: Glandular hyperplasia. The tissues were stained with haematoxylin-eosin stain

The formulations were fluid at room temperature and were rapidly transformed to viscous gels at 37°. Both gels showed initial burst followed by gradual release and permeation and release from C-GP gels were more rapid than from the gels with PEG (figs. 1 and 2). The gels had mucoadhesion comparable to or greater than chitosan at 25°, but the adhesiveness was significantly reduced on gelation at 37° (fig. 3). *In vivo* results revealed significant and sustained reduction in locomotor activity of mice on IN administration of both formulations in comparison to drug solution (fig. 4). Histopathology revealed minor damage to nasal tissues after 5 days of exposure (fig. 5).

The weakly basic GP prevents precipitation of chitosan on increase in pH and facilitates hydrophobic interactions on slight elevation of temperature resulting in thermoreversible systems. The sols showed good mucoadhesion but gelling reduced this effect due to stronger bonding within polymeric chains rather than with mucin. The pronounced and prolonged depression in locomotor activity of mice strengthens the hypothesis of direct delivery to brain. Preliminary studies also indicate a good safety profile. In conclusion, the developed biogel formulations could prove to be promising alternative therapy with RB. The formulations offer convenience of administration and prolong nasal residence time and thereby nasal absorption of RB.
